# A novel isolated phage targeting *Pseudomonas aeruginosa* demonstrates therapeutic potential

**DOI:** 10.1371/journal.pone.0349089

**Published:** 2026-05-13

**Authors:** Xinjie Wang, Zhao Zhang, Emanuela Garbarino, Shuhua Chen, Genyan Liu, Huamin Tang, Hua Xie

**Affiliations:** 1 Department of Immunology, National Vaccine Innovation Platform, School of Basic Medical Sciences, Nanjing Medical University, Nanjing, China; 2 Department of Critical Care Medicine, Changzhou Cancer Hospital, Changzhou, China; 3 Department of Laboratory Medicine, the First Affiliated Hospital with Nanjing Medical University, Nanjing, China; 4 The Laboratory Center for Basic Medical Sciences, Nanjing Medical University, Nanjing, China; 5 Jintan Hospital Affiliated to Jiangsu University, Changzhou, China; Sun Yat-Sen University, CHINA

## Abstract

*Pseudomonas aeruginosa* is a Gram-negative opportunistic bacterium responsible for severe infections such as pneumonia, septicemia, and keratitis. It poses a significant treatment challenge due to its extensive antibiotic resistance and its capacity to form biofilms, which provide bacterial communities with a protective barrier against antibiotics. An increasing number of studies have identified phage therapy as a potential therapeutic solution amidst the current crisis of antibiotic resistance in medicine. Here, we isolated three novel phages in the campus environment and selected one, PW01, for detailed analysis. Host range was determined against clinical isolates, and biological features were evaluated through growth kinetics and biofilm inhibition assays. Whole genome sequencing and annotation were conducted to confirm its lytic nature. *In vivo* efficacy was assessed using a murine wound infection model. PW01 displayed a relatively broad host spectrum and effectively suppressed bacterial growth *in vitro*. It disrupted established biofilms and showed genomic features consistent with a strictly lytic lifestyle. In mice, treatment with PW01 combined with antibiotics resulted in greater bacterial reduction compared with either treatment alone. These findings demonstrate that PW01 possesses both i*n vitro* and *in vivo* activity against *P. aeruginosa* and support the potential of phage-antibiotic combination therapy as an effective strategy against multidrug-resistant (MDR) infections.

## Introduction

*Pseudomonas aeruginosa* (*P. aeruginosa*) is an opportunistic Gram-negative bacterium named after the water-soluble green pigment produced during its metabolism, which leads to the green color of the wounds and traumas where it propagates [1,2]. This bacterium is ubiquitous in nature and a component of the normal microbial flora of human skin [3]. It also has been reported to be one of the most important pathogens of hospital-acquired infections, especially in infections of the urinary tract and respiratory tracts, where it can trigger various acute and chronic infectious diseases, including pneumonia, urethritis, septicemia, and keratitis [4]. In severe cases of physical trauma and burns, as well as in immunocompromised patients, it usually poses a significant threat, even leading to life-threatening infections [5]. The surge in nosocomial infections caused by *P. aeruginosa* that negatively impacts patients’ lives and healthcare costs require decisive measures to curb and eradicate these infections.

The current treatment for *P. aeruginosa* infections employs a range of antibiotics, including β-lactams, fluoroquinolones, tetracyclines, aminoglycosides, and polymyxins, among others [6]. However, there is clear evidence that the number of MDR *P. aeruginosa* strains has increased significantly over the past two decades, largely due to the inappropriate and excessive use of antibiotics, posing a serious clinical challenge [7]. As an aerobic bacterium, *P. aeruginosa* can withstand harsh environmental conditions and survive with minimal nutrients, earning it a place on the World Health Organization’s (WHO) Bacterial Priority Pathogens List (BPPL) as a high-priority pathogen [8]. Moreover, the pathogenicity of *P. aeruginosa* increases with the biofilm formation, a self-produced matrix of extracellular polymeric substances (EPSs) composed of metabolic products such as lipopolysaccharides, proteins, extracellular DNA (eDNA), and cellular debris, arranged to form a complex architecture that assures protection from external influences [9]. The biofilm provides a physical barrier to the penetration of antibiotics and other antimicrobial agents and sustains the bacterial life [10]. These factors have contributed to the rampant spread of *P. aeruginosa* in nosocomial infections, underscoring the urgent need for new antimicrobial and bacteriostatic strategies to combat these challenges [11,12].

The escalating difficulty in eradicating bacterial infections with antibiotics alone has led to a renewed interest in bacteriophage’s application as a potential solution against multidrug-resistant bacteria (MRB). Phages, a class of viruses parasitizing microorganisms, are abundant and highly diverse on Earth. They possess relatively small genomes, are widely distributed, and exhibit high specificity and self-limiting characteristics [13,14]. In particular, the high degree of specificity of phages, which lyses only certain types of target bacteria without posing a threat to mammalian cells, has highlighted their beneficial use as antimicrobial therapy since their discovery [15,16]. In recent years, with the emergence of drug-resistant bacteria and the slowdown in the introduction of new antibiotics, phage therapy has once again attracted researchers and clinicians’ attention [17].

Various studies have isolated and characterized *P. aeruginosa* phages from environmental, wastewater, and clinical sources. These include members of the *Myoviridae*, *Podoviridae*, and *Siphoviridae* families, as well as jumbo phages (genomes >200 kb) belonging to the order *Caudoviricetes*. Representative lytic phages like PA5oct and phiKZ have shown strong antibacterial and biofilm-degrading activities against *P. aeruginosa*, both individually and in combination as phage cocktails, highlighting their therapeutic potential [18–21].

Early, non-controlled human studies reported the direct application or ingestion of phages in different concentrations and/or dilutions [22,23]. Nowadays, the use of phage is commonly experimented in synergy with antibiotic therapy [24,25]. However, in mouse models, phage therapy alone has also produced positive results [26–28]. Since the efficacy of phage activity requires direct contact with their targeted bacteria, various strategies for phage delivery are under investigation [29–31].

In this study, our aim was to isolate and characterize bacteriophages targeting *P. aeruginosa,* particularly those obtained using antibiotic-susceptible *P. aeruginosa* strains isolated from natural environments, and to evaluate their potential as therapeutic agents against clinically relevant strains. Specifically, we sought to assess their lytic activity and spectrum, with a view toward their possible application in treating *P. aeruginosa*-associated infections.

We isolated three bacteriophages, designated PW01, PW02, and PW03. PW01, the primary focus of this study, was comprehensively characterized at both genomic and biological levels, whereas PW02 and PW03 were included to support antibacterial evaluation. PW01 is a newly identified lytic phage that exhibited strong in vitro activity and a relatively broad lytic spectrum against clinically relevant *P. aeruginosa* isolates, as well as efficacy in a mouse skin wound infection model. These findings highlight PW01 as a promising therapeutic candidate against *P. aeruginosa* infections.

## Materials and methods

### Bacterial strains and cultivation conditions

The host strain of *P. aeruginosa* was isolated from the soil samples collected from the Nanjing Medical University campus, and its identity was confirmed through ribotyping following the procedure from a previous study [32]. Briefly, a colony with metallic luster was cultured in liquid medium for genomic DNA extraction using the Bacterial Genome Extraction Kit (Tiangen). The 16S rRNA gene was amplified by PCR using the reported universal primers (5’-AGAGTTTGATCATGGCTCAG-3’ and 5’-TACGGTTACCTTGTTACGACTT-3’). The PCR product was sequenced (General Biol) and analyzed using NCBI BLAST (https://blast.ncbi.nlm.nih.gov/Blast.cgi). *P. aeruginosa* strains utilized in the study were cultivated in standard BHI medium or LB medium, or BHI-agar plates at 37 °C.

### Antibiotic susceptibility assay of *P. aeruginosa*

The antimicrobial susceptibility test of *P. aeruginosa* was performed in the Department of Laboratory Medicine, Jiangsu Maternal and Child Health Hospital, employing the VITEK 2 Compact for drug susceptibility identification, following the standard protocol from the Clinical and Laboratory Standards Institute (CLSI) [33].

### Phage isolation and purification

Bacteriophages that can infect the soil-sourced strain of *P. aeruginosa* were isolated from the sewage sample collected from Nanjing Medical University campus. Briefly, the sewage sample was firstly filtered through qualitative filter paper (Double ring) and centrifuged at 8000 × g for 10 min at 4 °C. The supernatant was further filtered using 0.45 µm pore size sterile membranes (Millipore). The filtrate was mixed with *P. aeruginosa* culture overnight at 37 °C for the enrichment of phages. After centrifugation, the supernatant was filtered through the membrane for further characterization. For plaque-formation testing, the enriched phage filtrate (100 µL) was mixed with an overnight *P. aeruginosa* culture (100 µL) and 4 mL of soft BHI agar, and then poured into a BHI-agar plate and incubated at 37 °C overnight. The next day, single plaques were picked up and immersed into 1.5 mL tubes containing SM buffer. The purification procedure was repeated three to four times until the formed plaques were of the similar size. To obtain high titer of bacteriophages, 150 mL of bacteriophage lysates were filtered through a 0.22 µm syringe filter (Millipore) and mixed with the same volume of 10% polyethylene glycol 8000 (PEG8000; Biosharp) by slowly rotating overnight. The mixture was centrifuged at 10,000 × g for 10 min at 4 °C and the pellet was re-suspended in SM buffer. The solution was then mixed with chloroform, followed by centrifugation at 10,000 × g for 10 min at 4 °C, and the supernatant was stored at 4 °C.

### Phage host-range assay

A spot test was performed to investigate bacterium susceptibility to phage infection [34]. The MDR *P. aeruginosa* strains used in this study as well as the reference strain *P. aeruginosa* ATCC 27853 were from the Bacterial Bank of the Laboratory Department of Jiangsu Maternal and Child Health Hospital. Briefly, the overnight-cultured bacteria (100 µL) were mixed with 4 mL of soft BHI medium and poured onto a BHI solid plate, cooled to room temperature, followed by dropwise adding of 5 µL of phage supernatant (about 10^9^ PFU/mL). After drying, the plates were incubated at 37 °C overnight.

### Bacteriophage morphology observation by transmission electron microscopy (TEM)

The bacteriophage morphology was analyzed by TEM. 10 µL of purified bacteriophage (about 10^10^ PFU/mL) was applied to a copper grid with a carbon support film, air-dried, and stained with 2% phosphotungstic acid negative stain solution. After 10 min, the stain was washed off with tap water and the sample was left to dry before being observed with a TEM (Thermo Scientific Helios 5 Hydra Dual Beam).

### Bacteriophage genome extraction, sequencing, and bioinformatics analysis

The bacteriophage genomic DNA was extracted using the Virus DNA/RNA Extraction Kit (Tiangen) and sequenced using Illumina NovaSeq paired-end platform (Personalbio). Firstly, A5-MiSeq and SPAdes were used to assemble the sequencing data and construct the contig [35,36]. Then the high-sequencing depth sequences were extracted and blasted against the NT libraries (Non-Redundant Protein Sequence Database) of NCBI to identify phage genome sequences. The results obtained by above software were analyzed using MUMmer (ANIm) for covariance analysis to determine the positional relationship between contigs and fill gaps. Finally, the results were corrected by Pilon software to obtain the final phage genome sequences [37,38].

### Optimal multiplicity of infection (MOI) detection and one-step growth curve determination

The overnight culture of *P. aeruginosa* was 1:100 diluted for further culture for 2 h at 180 r/min at 37 °C until the logarithmic growth stage (OD_600_ = 0.6, about 4.2 × 10^7^ CFU/mL). Then the bacterial culture was transferred into new tubes with serial ten-fold dilutions of phages, cultured at 37 °C for about 3.5–4 h at 180 r/min, and centrifuged at 10,000 × g for 10 min. The supernatant was collected for phage titration using double-layer agar plate method. The highest titer was considered as the optimal MOI.

The bacterial solution (about 4.2 × 10^7^ CFU/mL) was mixed with the bacteriophage lysate at MOI of 10, incubated at 37 °C for 15 min, followed by centrifugation at 10,000 × g for 30 s. The pellet was washed twice with 1 mL of BHI medium, resuspended, and cultured at 37 °C with constant shaking at 180 r/min. Every 10 min, 50 µL of sample was harvested and centrifuged at 10,000 × g for 30 s. The phage titers in the supernatant were determined by the double-layer agar plate method. The results of the host bacterium without phage and the phage without host bacterium were used as control. The one-step growth curve was plotted with the infection time as the horizontal coordinate and the phage titer in the infection system as the vertical coordinate to determine the latent period, burst period and outbreak amount of the phage.

### Bacterial reduction assay

*In vitro* bacterial reduction experiment was assayed in a 24-well plate, each containing BHI medium and a soil-derived *P. aeruginosa* culture, and were kept at 37 °C until the OD_600_ measured as 0.2 (about 1.2 × 10^7^ CFU/mL). Then the experimental groups were treated with a single phage or combined. Phage doses were calculated based on the optimal MOI of 0.01. In the combination group, PW01 and PW02 were mixed at equal volumes, each contributing half of the total phage amount. Meanwhile the negative control group was treated with an equivalent volume of BHI medium. The 24-well plate was kept at 37 °C, and OD_600_ readings were taken every 1 h for up to 10 h with a microplate reader (BioTek Synergy MX).

### Bacterial biofilm removal assay

The overnight culture of soil-derived *P. aeruginosa* was hundredfold dilution with LB medium and then shaken at 37 °C for about 3 h until the logarithmic growth phase. Subsequently, 500 µL of bacterial solution (about 4.2  × 10^7^ CFU/mL) and 500 µL of LB medium were added to the 24-well plates and incubated at 37 °C under static condition for 24 h. Then each well was washed twice with sterile PBS, and then the culture medium was added. The phage solution (at a concentration of 10^8^ PFU/mL) or the same volume of LB medium was added, and then the plates were incubated at 37 °C. After 4 or 8 h of culture, the plates were harvested, and the culture supernatant was aspirated. Each well of the plates was washed three times with PBS and then stained with 0.1% crystal violet dye for 10 min, followed by washing with tap water. The stained biofilm was dissolved in 95% ethanol and measured at OD_590_ using the Microplate reader (BioTek Synergy MX).

### Wound infection and phage therapy

All protocols in this study were approved by the Committee on the Ethics of Animal Experiments of Nanjing Medical University (approval number: 2411001). Six-week-old female BALB/c mice were randomly divided into four groups (n = 3 per group): control (PBS), kanamycin-treated, phage-treated, and combination-treated (kanamycin + phage). Mice were anaesthetized with 20% urethane (Sigma-Aldrich). After hair removal and disinfection, a sterile biopsy punch (5-mm diameter) was used to punch through the full thickness of the back skin below the shoulder blades of mice. Then the soil-derived *P. aeruginosa* (1500 CFU bacteria resuspended in 5 µL of PBS) was topically applied to each wound to establish infection. Mice were housed in individual cages to prevent cross-contamination or interaction. After 18 h, treatments were administered topically. The kanamycin group received kanamycin at a concentration of 0.5 mg/mL, while the phage group received 5 µL of PW01 suspension (10^9^ PFU per dose). The combination group received both treatments, and the control group received an equal volume of PBS. Kanamycin was selected as a representative antibiotic based on its routine use in our laboratory for antimicrobial susceptibility testing against *P. aeruginosa*. Mice were monitored for approximately 12 h after treatment. At the experimental endpoint, mice were humanely euthanized by cervical dislocation under anesthesia. Wound tissues were aseptically excised, homogenized using magnetic beads, and serially diluted for bacterial enumeration on LB agar plates. The survival status of mice was continuously monitored for 2 days.

### Statistical analysis

The statistical analysis of data was presented as means ± standard deviation (SD). All experiments were performed with at least three independent biological replicates (n = 3). For the bacterial reduction assay, differences among groups over time were analyzed using two-way analysis of variance (ANOVA) followed by Dunnett’s multiple comparisons test, with each treatment group compared to the control. For the bacterial biofilm removal assay, data were analyzed using two-way ANOVA followed by Sidak’s multiple comparisons test. For the wound infection and phage therapy experiment, differences in bacterial burden among groups were analyzed using one-way ANOVA followed by Tukey’s multiple comparisons test. Prism 10 software was used, and a *P-*value less than 0.05 (*P* < 0.05) was accepted as statistically significant.

## Results

### A soil-derived strain of *P. aeruginosa* is susceptible to a set of antibiotic drugs

*P. aeruginosa* was isolated from fresh soil leachate using streak plate method. We found single colonies with metallic luster. Identification of bacterial strain was carried out by molecular typing. About 1500 bp DNA fragments were amplified using universal 16S rRNA primers through PCR, followed by sequencing. The sequence data was aligned with the NCBI database using BLAST, showing 100% identity with the 16S rRNA gene of *P. aeruginosa*. The antibiotic susceptibility experiment assay showed that the soil-derived strain of *P. aeruginosa* was susceptible to a range of antibiotic drugs empirically used in clinical practice (Table 1). Therefore, this soil-derived strain of *P. aeruginosa* was relatively safe (classified under biosafety level 2 [39]) and well-suitable for subsequent phage-isolation experiments.

**Table 1:** Susceptibility of the soil strain *P. aeruginosa* to different antibiotics.

Antibiotic	MIC (µg/mL)	Interpretation (CLSI)
Piperacillin/Tazobactam	8	+
Ceftazidime	2	+
Cefepime	2	+
Imipenem	2	+
Meropenem	1	+
Amikacin	≤2	+
Ciprofloxacin	≤0.25	+
Levofloxacin	0.25	+
Cefoperazone/Sulbactam	≤8	+
Colistin	2	+
Tobramycin	≤1	+
Ticarcillin/Clavulanic Acid	16	+
Minocycline	8	–
Doxycycline	8	–

+ indicates susceptible; – indicates resistant, according to CLSI guidelines

### Isolation of Three phages from a university campus

Different from traditional phage isolation methods that rely on pathogenic bacteria from clinical sources, we used the soil-derived strain as the host for isolating phages, which may complement previous trials. After several attempts, three phages were successfully isolated from the university sewage. The phages were sequentially designated in order of their discovery as PW01, PW02, and PW03 and were further evaluated for their lytic effects on the clinical strains of *P. aeruginosa* and ATCC standard strain 27853.

### The phage PW01 displays the broadest host spectrum

Employing Spot test with the ATCC standard strain and MDR *P. aeruginosa* strains isolated from different patients kept in the clinical specimen bank of the Department of Laboratory of Jiangsu Provincial People’s Hospital, we assessed the phage spectrum. The ability to form lytic spots indicates that the phage has lysed the targeted bacteria strain, and the clarity and translucency of the lytic spots indicate their lysing capacity. Results showed that PW01 had a broader lysis spectrum and stronger lysis capacity compared to PW02, and PW03 (Table 2) and indicated that phage PW01 is a more promising candidate phage for a wider range of applications. Therefore, further exploration was focused on PW01.

**Table 2:** Lytic activity of the three phages on different *P. aeruginosa* strains.

Host No.	Characteristics of Host strain	Infectivity of PW01	Infectivity of PW02	Infectivity of PW03
Pa001 Pa002 Pa003 Pa004 Pa005 Pa006 Pa007 Pa008 Pa009 Pa010 Pa011 Pa012 Pa013 Pa014 Pa015 Pa016 Pa017 Pa018 Pa019 Pa020 Pa021 Pa022 Pa023 Pa024 Pa025 Pa026 Sum	MDRP CRPA CRPA CRPA CRPA MDRP MDRP CRPA CRPA CRPA CRPA CRPA CRPA CRPA ATCC 27853 Soli Strain	++ ++ ++ + ++ ++ ++ ++ + ++ ++ + ++ – + ++ + + + – ++ ++ ++ ++ ++ ++ 24	++ ++ ++ – – – ++ – + ++ ++ + ++ ++ – ++ + – + – ++ ++ ++ + ++ ++ 19	++ ++ ++ – – – ++ ++ – ++ ++ – + ++ – + – – + – – ++ ++ – ++ ++ 15

++: plaque clear; + : plaque cloudy; -: No plaque

MDRP: multidrug resistant *P. aeruginosa*; CRPA: carbapenem-resistant *P. aeruginosa*

### Phage PW01 is a member of the *Myoviridae* family

**Fig 1:**
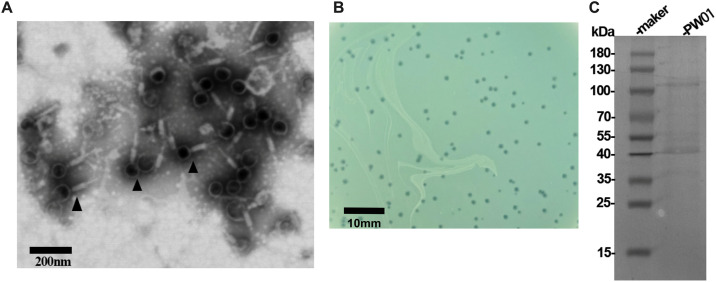
Characterization of purified PW01. (A) Transmission electron micrographs of the purified PW01 phage. Purified phages were fixed, stained and observed under a transmission electron microscope for morphology analysis. The black arrows highlight individual phages as representative samples. Scale bar, 200 nm. (B) Plaques of PW01 with distinct boundaries on a double-layer agar plate. (C) Protein electrophoresis followed by Coomassie Blue staining shows the size and distribution of the major coat proteins of phage PW01.

TEM analysis revealed that PW01 phage possessed an isometric head structure with a diameter of 70 nm (Fig 1A). The contractile tail, which consisted of a neck, a contractile sheath, and a central tube, measuring 120 nm in length. According to the International Committee on Taxonomy of Viruses (ICTV), bacteriophage PW01 should be classified into the *Myoviridae* family in the order *Caudovirales*. The plaques of PW01 ranging from 0.5 to 1.0 mm in diameter had well-defined boundaries which were highly probably produced by lytic phages (Fig 1B). After phage concentration and purification, by SDS polyacrylamide gel electrophoresis and staining, we were able to observe that the major coat proteins of the phage were at approximately 110 kDa and 40 kDa (Fig 1C).

### Genome isolation and phylogenetic analysis of Phage PW01

**Fig 2:**
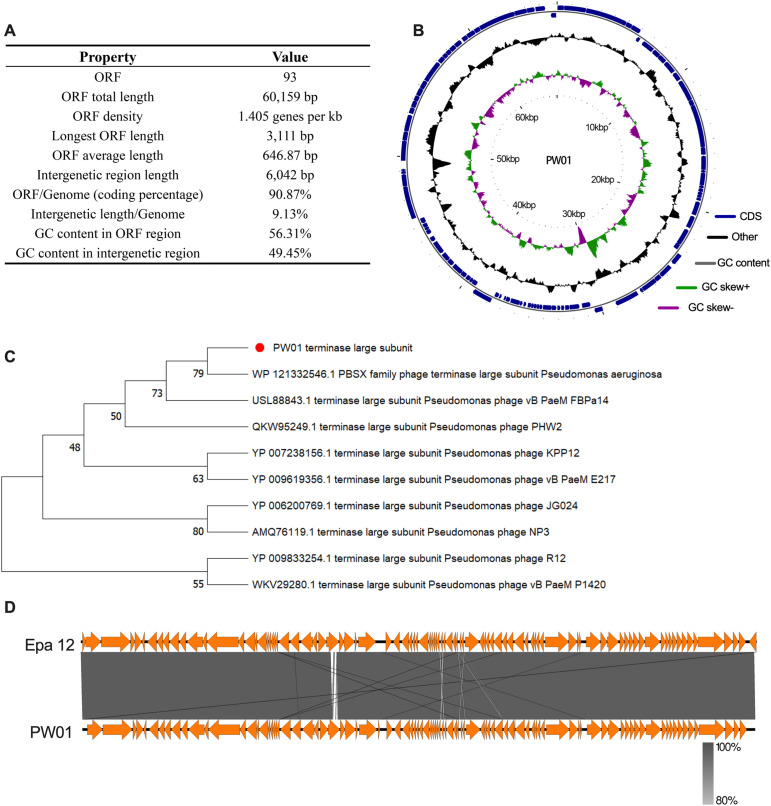
Genomic analysis of PW01. (A) The complete genomic DNA sequence was deduced through data filtering and de novo assembly, followed by open reading frame analysis. A total of 60 kb of genomic sequence was identified, with the comprehensive ORF information meticulously detailed. (B) The integrated genome sequence, gene prediction and non-coding RNA prediction were formatted into a standard GBK (GenBank) file, depicted as a circular diagram of PW01. (C) The neighbor-joining tree illustrates the phylogenetic relationship between the terminase large subunits of PW01 and related Pbunavirus genus. The red dot represents PW01, with node numbers representing the bootstrap probabilities. (D) Comparative genome alignment of phage PW01 shows its evolutionary relationship with the Pseudomonas phage Epa12 (MT118291.1).

The whole genome of the PW01 phage was a double-stranded DNA, with sequencing confirming an approximately 66 kb and a G + C content of 55.68% (Fig 2A). Analyzing the PW01 genome sequence on PhageTerm software suggested that the PW01 phage has a circular genome (Fig 2B), and the genomic annotation information has been submitted to GenBank to obtain the accession number: PV189960. Further investigation revealed that there were no genes encoding tRNAs in the phage genome, suggesting that the protein synthesis of this phage was entirely dependent on the host’s tRNA machinery. Annotation of phage PW01 sequence revealed the presence of 93 putative open reading frames (ORFs). Based on the conservatism of the large subunit of the terminal enzyme, approximately twenty phages with close affinity were retrieved by blast to plot the genetic evolution tree as shown in Fig 2C. It suggests that PW01 belongs to the *Pbunavirus LS1* genus. A strain of phage Pseudomonas phage Epa12 (accession number: MT118291.1), which is close to its relatives, was selected for covariance analysis by Easyfig software (Fig 2D), which clearly showed the corresponde‌‌nce between the CDS regions of the two phages.

### Optimal multiplicity of infection (MOI) and one-step growth curve of PW01

To determine the optimal MOI, we mixed the phage PW01 with its host *P. aeruginosa* at various ratios. The bacterial suspension used for infection had an optical density at 600 nm (OD₆₀₀) of 0.6, corresponding to approximately 4.2 × 10^7^ CFU/mL. The highest phage output was achieved at an MOI of 0.01, with a mean titer of 1.87 × 10^10^ PFU/mL. In contrast, infections at higher MOIs (0.1, 1, 10, and 100) yielded lower final titers, all in the range of 6.8 × 10^9^ to 1.1 × 10^10^ PFU/mL, suggesting the occurrence of premature lysis under these conditions. The phage production at MOI = 0.001 was significantly lower (5.83 × 10^8^ PFU/mL), indicating insufficient initial infection. Therefore, an MOI of 0.01 was identified as optimal for efficient PW01 propagation and was used in subsequent experiments.

**Fig 3:**
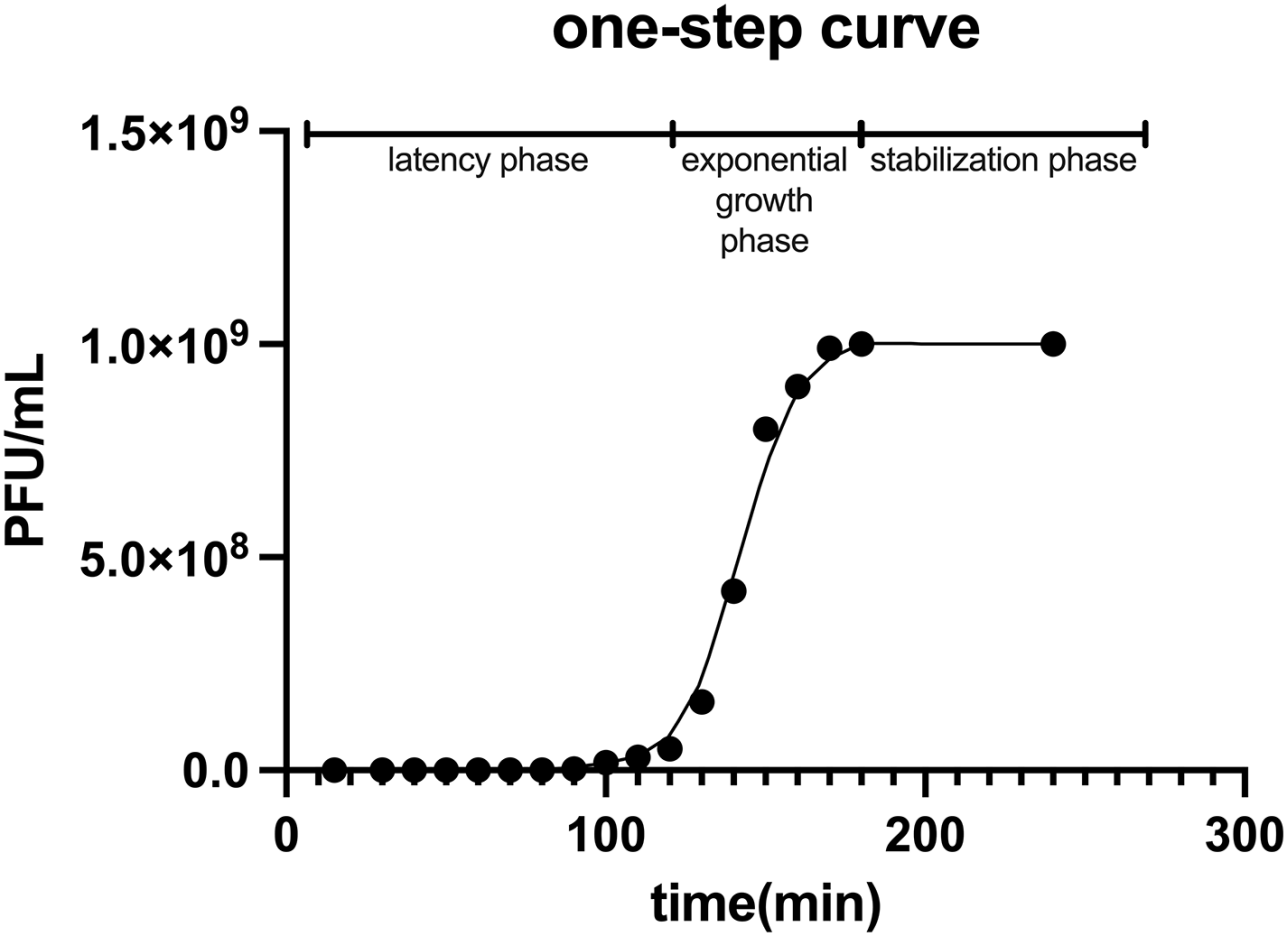
One-step growth of PW01. *P. aeruginosa* was infected with PW01 at the MOI of 10, and samples were harvested and centrifuged at 10 min intervals. The supernatants were serially diluted and spread on BHI agar plates to quantify free phages at every time point. The latency period, exponential growth phase, and stabilization phase were elucidated.

Based on the optimal MOI of PW01, we explored its one-step growth curve to quantitatively characterize the growth pattern of this phage and evaluate its infection potential. We performed the assay with an initial MOI of 10 to ensure synchronous infection, and the results are shown in Fig 3. The phage titers remained stable during the first 80–90 min (eclipse period), followed by a sharp rise between 90 and 170 min, reaching a plateau at approximately 1.0 × 10⁹ PFU/mL by 180 min. The latent period was estimated to be around 90 min, and the burst size was calculated to be about 20 PFU per infected cell. The burst period of phage replication lasted about 80 min and then the phage titer remained relatively stable, indicating a stabilization phase (Fig 3). These findings suggested that the PW01 phage is characterized by a short incubation period and a high virulence. The raw data for spot tests and CFU counts are provided in S2–S4 Tables.

### Phage PW01 can inhibit *P. aeruginosa* growth *in vitro*

**Fig 4:**
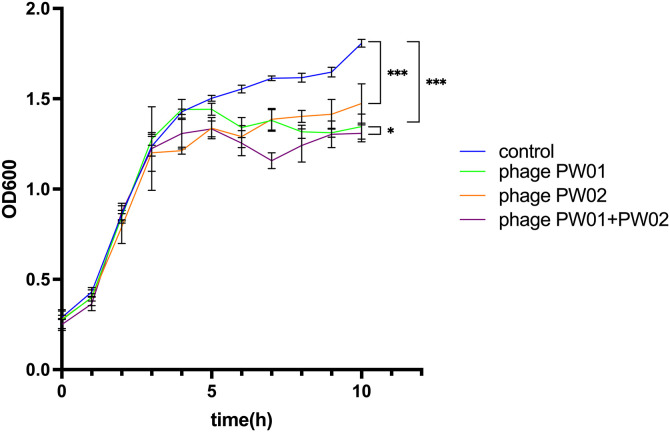
PW01 effectively inhibits *P. aeruginosa* growth. The lytic effect of the phage PW01, phage PW02, and their combined application on the growth of *P. aeruginosa* in pre-logarithmic phase was assessed in comparison to the control without phage. Measurements were performed at 1 h intervals, and all time points, including 3 h and 4 h, are included in the dataset. D*ata* are shown as mean ± SD (n = 3). Statistical analysis was performed at the 10 h time point using two-way ANOVA followed by Dunnett’s multiple comparisons test. *P < 0.05; **P < 0.01; ***P < 0.001.

The inhibitory effect of phage PW01 on soil-derived *P. aeruginosa* became evident after 4 h (Fig 4), when the OD₆₀₀ value decreased from 1.43 ± 0.01 in the control group to 1.34 ± 0.06, representing a 13.6% reduction (*P* < 0.05). In contrast, phage PW02 showed an earlier lytic effect after 3 h (OD₆₀₀ = 1.20 ± 0.11 vs. control = 1.24 ± 0.06), but its overall inhibitory capacity gradually weakened at later stages (1.47 ± 0.11 at 10 h, 19.0% lower than the control, *P* > 0.05). The combined application of PW01 and PW02 exhibited the most potent inhibitory effect, with OD₆₀₀ reaching 1.31 ± 0.05 at 10 h—approximately 27.6% lower than the control (*P* < 0.01). Beyond this time point, no further decrease in absorbance was observed, suggesting that bacterial lysis had reached completion, and the residual absorbance corresponded to cell debris. These findings indicate that the phage cocktail demonstrates stronger and more sustained antibacterial activity than single-phage treatments.

### Phage PW01 can dissolve *P. aeruginosa* biofilm

**Fig 5:**
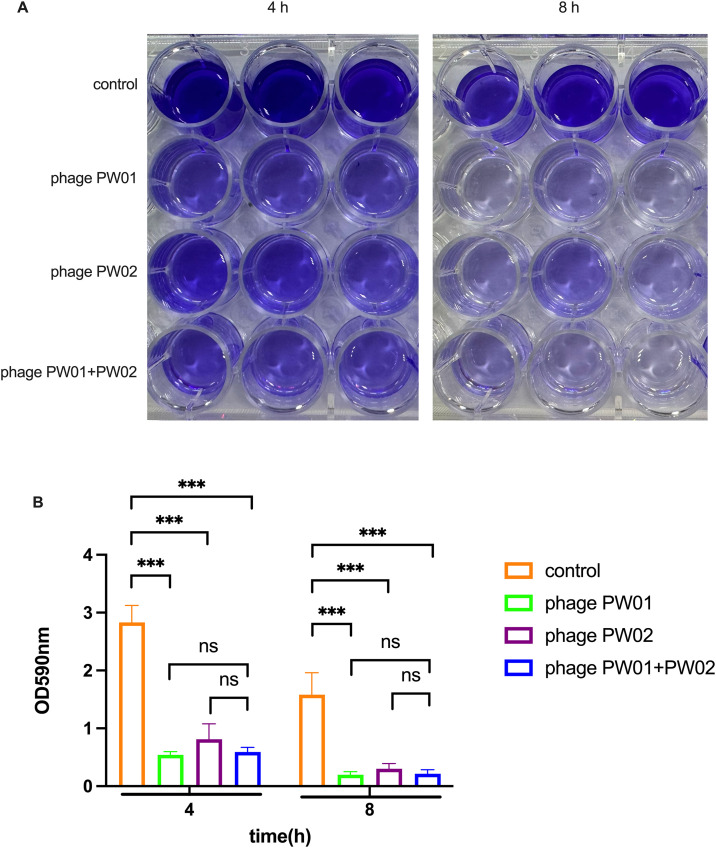
PW01 efficiently dissolves *P. aeruginosa* biofilm. (A) *P. aeruginosa* biofilm formed in 24-well plates under static culture condition, then various phage solutions were added into each cell and incubated for another 4 or 8 h. The remaining biofilms were washed with PBS and stained with crystal violet. (B) The OD_590_ absorbance was measured to quantify the remaining biofilm. Data are presented as mean ± SD (n = 3). Statistical significance was determined using two-way ANOVA followed by Sidak’s multiple comparisons test. *P < 0.05; **P < 0.01; ***P < 0.001.

We tested biofilm removal by measuring the OD values after biofilm formation. We picked the maximum stability of biofilm formation time (4h) and the gradual disintegration time (8h) to measure the OD_590_ absorbance after staining of crystal violet (Fig 5A). At 4 h, the OD₅₉₀ value of the control group was 2.83 ± 0.29, whereas that of the PW01-treated group was 0.54 ± 0.06, representing an 80.9% reduction (*P* < 0.01). The PW02 treatment resulted in a smaller reduction (0.81 ± 0.27, 71.4% reduction), while the combination of PW01 and PW02 achieved a comparable effect (0.59 ± 0.08, 79.1% reduction). At 8 h, the control group maintained an OD₅₉₀ of 1.58 ± 0.38, whereas PW01 and the phage cocktail both reduced it to approximately 0.20 ± 0.05 and 0.21 ± 0.06, respectively (both > 86% reduction, *P* < 0.01). PW02 alone showed weaker removal efficiency (0.30 ± 0.09, 81.0% reduction). The statistical analyses revealed that phage PW01 had a stronger removal capacity of biofilm formed by soil-derived *P. aeruginosa* strain than the phage PW02. However, there were no significant differences between PW01 and the combination of PW01 and PW02 (Fig 5B), which demonstrated that although the combination of the two phages was able to significantly inhibit the growth of *P. aeruginosa in vitro*, the result was different in the biofilm removal assay.

### Phage PW01 effectively cures the skin wound infection

**Fig 6:**
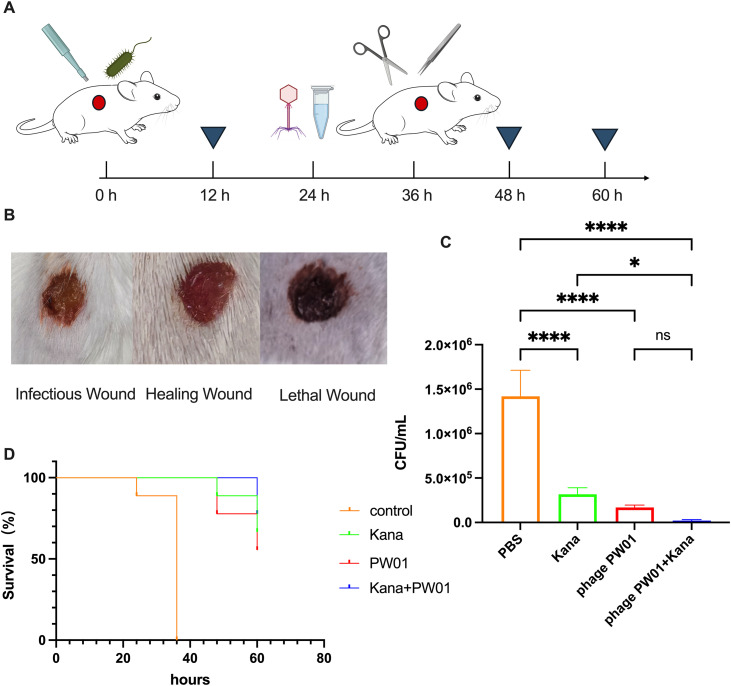
Different treatments of *P. aeruginosa* infection in mouse skin model. (A) Six-week-old female BALB/c mice were anaesthetized. A sterile biopsy punch (5-mm diameter) was used to create full-thickness wounds of the back skin below the shoulder blades of mice. Then the *P. aeruginosa* was applied to each wound. After 18 h, the mice were treated with kanamycin, phage or combined, and PBS as a control. (B) The representative images are as follows: the left panel displays a status of infection; the central panel illustrates a healed wound; and the right panel depicts a fatal infectious wound. (C) Colony-forming units of bacteria after different treatments were enumerated. Skin tissues were isolated, homogenized, diluted 100-fold, and plated on ampicillin agar to count *P. aeruginosa* colonies. Data are presented as mean ± SD (n = 3). Statistical significance was calculated using one-way ANOVA followed by Tukey’s post hoc test. *P < 0.05, **P < 0.01, ***P < 0.001, ****P < 0.0001. (D) The overall survival curves of mice with skin infections within different treatment groups were plotted. The survival of mice continued to be observed after bacteria colonies were counted and finally plotted as a percentage of survival.

To evaluate the *in vivo* efficacy of phage PW01, we established a murine skin infection model. After 18 h post-infection, wounds exhibited varying degrees of abscess formation and exudation depending on the treatment (Fig 6A). The PBS-treated mice showed severe purulent exudation, while the kanamycin- and phage-treated groups exhibited reduced inflammation. Notably, mice receiving the combination of PW01 and kanamycin displayed dry wound surfaces without exudation and early scab formation (Fig 6B).

Quantification of bacterial loads revealed that phage PW01 alone reduced the viable bacterial count by 76.6% compared with the PBS control (1.42 × 10⁶ ± 2.9 × 10⁵ vs. 3.33 × 10⁵ ± 7.2 × 10⁴ CFU/mL, *P* < 0.01), whereas the combination of PW01 and kanamycin achieved a 98.6% reduction (2.25 × 10⁴ ± 1.2 × 10⁴ CFU/mL, *P* < 0.001) (Fig 6C). Bacterial load correlated positively with the severity of wound exudation, confirming that higher CFU counts were associated with more severe infections.

Survival analysis further demonstrated that all mice in the PBS group died within 24 h, while most mice in the other treatment groups survived (Fig 6D). Collectively, these results indicate that phage PW01 exhibits substantial therapeutic potential against *P. aeruginosa* skin infections, particularly when used in synergy with kanamycin.

## Discussion

The advent of antibiotics in the early twentieth century revolutionized the treatment of infectious diseases, significantly reducing associated morbidity and mortality caused by infectious diseases. However, the improper use and abuse of these drugs have resulted in an impasse against bacterial infections with the surge of MDR bacterial strains. The increase in nosocomial infection caused by the MDR *P. aeruginosa* has highlighted the urgent need to develop alternative strategies to conventional antibiotic therapy. Bacteriophages, one of the earliest treatments, are now being reevaluated in the fight against bacterial infections. Ubiquitous in nature, they have the capacity to selectively eradicate bacterial colonies. Thus, they can be considered natural antibacterial agents that efficiently suppress the rampage of drug-resistant bacteria strains and bring significant therapeutic benefits [40,41].

Since *P. aeruginosa* is one of the major pathogens involved in wound infection, the importance of an alternative and/or complementary strategy for MDR strains is urgently needed. In this study, the environmental-derived strain of *P. aeruginosa* was isolated and used as the host. Three novel specific phages were isolated from the university sewage and designated PW01, PW02, and PW03. They were able to form translucent and neatly edged phage spots when cultured on double-layer agar plates, which showed typical characteristics of lytic phages. When applied to MDR *P. aeruginosa* strains obtained from clinical patients, phage PW01 showed the highest lysis rate on almost all 24 tested bacteria strains. Compared with the previously reported N4-like phages DSS3Φ2 and EE36Φ, which have latent periods of 3 h and 2 h, respectively, PW01 exhibited a shorter latent period, enabling it to exert lytic activity more rapidly [42,43]. The biological characteristics of PW01, including its short latency, strong lytic activity, and potential synergy with antibiotics, highlight its promise as a therapeutic candidate. Overall, these results indicate promising clinical application for the treatment of skin wounds infected with antibiotic-resistant strains of *P. aeruginosa.*

Biofilm formation of *P. aeruginosa* is a crucial obstacle in curing bacterial infections. Thus, we tested the capability of the phages to reduce the biofilm formation and facilitate their penetration through this protective barrier to directly attack and lyse bacteria. Indeed, our results revealed that PW01 can effectively lyse the host and remove the biofilm. The closer connection to the *in vivo* situation was shown by testing PW01 effects in healing of mouse wounds infected with *P. aeruginosa*. The results confirmed the beneficial effects of PW01 treatment alone or in combination with kanamycin, resulting in a reduction of the bacterial colony numbers in the wounds, complete wound healing, and an improved long-term survival rate of mice. Interestingly, although the number of colonies of *P. aeruginosa* surviving in the phage PW01-treated group was slightly lower than that in the kanamycin-treated group, the overall treatment was slightly inferior in terms of the survival curve of the mice. It is possible that the lysis of *P. aeruginosa* by phage PW01 causes a dramatic release of endotoxin and other bacterial components, eliciting a more pronounced immune response combined with a traumatic shock, which is more likely to result in death.

These data establish a sufficient theoretical foundation for the subsequent development of antimicrobial agents based on PW01 phage and indicate promising clinical applications for the treatment of skin wounds infected with antibiotic-resistant strains of *P. aeruginosa*. However, this study also has some limitations. In terms of exploring biological characteristics, we could further refine more conditions to explore the phage’s resistance to different living environments, its lytic capacity, and the efficiency of progeny phage production. In the mouse experiments, we did not test other types of antibiotics, nor did we consider the frequency of treatment inoculation. Modifying these conditions might more thoroughly eliminate bacteria or could also induce the production of anti-phage antibodies in mice, potentially weakening their lytic capacity. Furthermore, to administer phages via injection, the concentration of the phages and the removal of endotoxins must be considered together with the observation of therapeutic impact on the infection process and survival status of mice.

## Supporting information

S1 Fig Raw image for Fig 1C. The original, uncropped Coomassie Brilliant Blue-stained SDS–PAGE gel, which corresponds to Figure 1C, displays the size and distribution of the major coat proteins of phage PW01. Two additional lanes on the right correspond to unrelated samples that were not included in the analysis.
(TIF)

S2 Table Optimal multiplicity of infection. Bacteriophage titers (PFU/mL) were determined at different multiplicities of infection (MOI). Each replicate represents an independent infection experiment performed under identical conditions (n = 3). The optimal MOI was identified based on the highest mean phage yield after infection of Pseudomonas aeruginosa.
(XLSX)

S3 Table Bacterial counts. The bacterial counts of the soil-derived *P. aeruginosa* culture were determined at different OD₆₀₀ values. Each replicate represents an independent bacterial culture (n = 3). At the end of the one-step growth curve, no viable colonies were detected after incubation at 37 °C for 12 h.
(XLSX)

S4 Table Wound infection and phage therapy. Bacterial loads (CFU/mL) were measured in wound infection models that were treated with PBS, phage PW01 alone, or phage PW01 combined with kanamycin (Kana). Each replicate represents an individual mouse wound sample (n = 6). Values represent bacterial counts after treatment for 12 h.
(XLSX)

S5 Text 16SrRNA sequencing result of the soil-derived *P. aeruginosa.* (TXT)

## Abbreviations

COPD: chronic obstructive pulmonary disease
MDR: multidrug-resistant
ILD: interstitial lung disease
BHI: brain heart infusion
SM buffer: 50mM Tris-HCl (pH7.5), 100mM NaCl, 8mM MgSO4, 0.01% Gelatin
PEG8000: polyethylene glycol 8000
PFU/mL: plaque-forming units per milliliter
TEM: transmission electron microscopy
PBS: phosphate-buffered saline
MOI: multiplicity of infection.
