# Supplementary figures and images for: A novel isolated phage targeting Pseudomonas aeruginosa demonstrates therapeutic potential
Source: PLoS One. 2026 May 13;21(5):e0349089. doi: 10.1371/journal.pone.0349089 (PMC13170885; doi:10.1371/journal.pone.0349089)

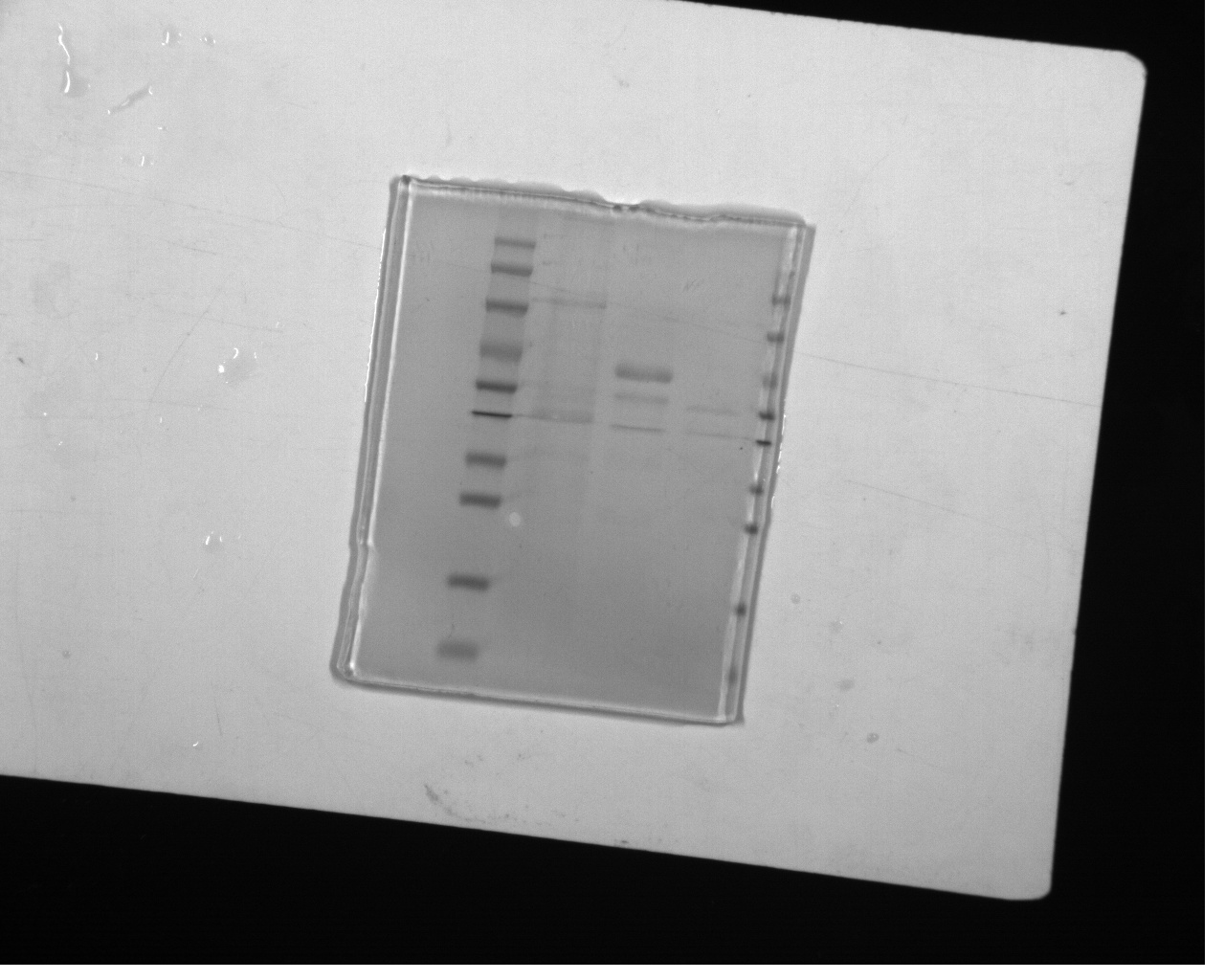

Supplement: S1 Fig — The original, uncropped Coomassie Brilliant Blue-stained SDS–PAGE gel, which corresponds to Figure 1C, displays the size and distribution of the major coat proteins of phage PW01. Two additional lanes on the right correspond to unrelated samples that were not included in the analysis. (TIF) [file pone.0349089.s001.tif]
